# A Multimodal Analysis Combining Behavioral Experiments and Survey-Based Methods to Assess the Cognitive Effect of Video Game Playing: Good or Evil?

**DOI:** 10.3390/s20113219

**Published:** 2020-06-05

**Authors:** Ji Hyeok Jeong, Hyun-Jung Park, Sang-Hoon Yeo, Hyungmin Kim

**Affiliations:** 1 Center for Bionics, Biomedical Research Institute, Korea Institute of Science and Technology, Seoul 02792, Korea; t20193@kist.re.kr (J.H.J.); phj@kist.re.kr (H.-J.P.); 2 School of Sport, Exercise and Rehabilitation Sciences, University of Birmingham, Birmingham B15 2TT, UK

**Keywords:** internet gaming disorder, video game addiction, impulsivity, response inhibition, prosaccade, antisaccade

## Abstract

This study aims to bridge the gap between the discrepant views of existing studies in different modalities on the cognitive effect of video game play. To this end, we conducted a set of tests with different modalities within each participant: (1) Self-Reports Analyses (SRA) consisting of five popular self-report surveys, and (2) a standard Behavioral Experiment (BE) using pro- and antisaccade paradigms, and analyzed how their results vary between Video Game Player (VGP) and Non-Video Game Player (NVGP) participant groups. Our result showed that (1) VGP scored significantly lower in Behavioral Inhibition System (BIS) than NVGP (*p* = 0.023), and (2) VGP showed significantly higher antisaccade error rate than NVGP (*p* = 0.005), suggesting that results of both SRA and BE support the existing view that video game play has a maleficent impact on the cognition by increasing impulsivity. However, the following correlation analysis on the results across individual participants found no significant correlation between SRA and BE, indicating a complex nature of the cognitive effect of video game play.

## 1. Introduction

On 24 May 2019, the committee of the World Health Organization (WHO) unanimously agreed to include video game addiction in the 11th edition of the International Classification Diseases (ICD-11) as a disease. WHO claimed that this decision is based on reviews of the available evidence and reflects a consensus of experts from different disciplines and geographical regions [1]. However, the decision has provoked substantial debates on whether video gaming should be devalued as a harmful or addictive activity, like gambling, that potentially leads to disease.

As pointed out by the WHO, there are studies emphasizing the addictiveness of video gaming and resulting health problems [2,3,4]. The Internet Gaming Disorder (IGD) group may experience health issues such as sleep deprivation, impulsivity and emotional disorders [5,6,7], and can be associated with neuroticism, low extraversion, and decreased conscientiousness [8,9]. On the other hand, opponents of WHO’s decision argue that there is a significant lack of scientific evidence or clear consensus defining symptoms of gaming addiction [10,11,12,13,14,15] and that this seemingly hasty decision could result in major social problems caused by faulty diagnoses and treatments, or by devastating impacts on the video game and related industries. Furthermore, they claimed that normal video game players could in fact benefit from several positive effects of video gaming, including cognitive enhancement or stress relief [16,17].

From a methodological point of view, studies on the psychological effects of video game playing can be largely divided into two groups: (1) Self-Report Analysis (SRA) studies and (2) Behavioral Experiment (BE) studies. The first group of studies mainly incorporates self-report surveys that are widely used in psychiatric [5,8,9,18,19,20,21,22,23,24,25] and behavioral studies [26,27,28,29], and aims to quantify how video game affects the quality of life. As far as we know, the results of such studies unanimously suggest that video game players score significantly worse in psychiatric scales, such as Brief Self-Control Scale (BSCS) [20,21,23] and Barratt Impulsiveness Scale (BIS-11) [19,25], indicating that video game players develop addictive, compulsive and antisocial behaviors that can potentially reduce the quality of life.

On the other hand, the second group of studies is mainly focused on how video game changes the cognitive performance factors related to perception, memory, attention, and sensorimotor control. Unlike the self-report-based studies mentioned above, this line of studies primarily takes quantitative, behavioral experiment, using psychometric measures such as sensory acuities [30,31,32], attention and memory capacities [33,34,35,36] or reaction times [37,38], in order to quantify the influences of video gaming on cognitive abilities. Interestingly, the results of such studies tend to advocate the efficacy of video gaming to improve cognitive performance and mental health. In particular, a series of studies have shown that video gaming has a positive effect on top-down inhibition and cognitive performance [39,40,41,42,43,44,45], which exhibits a sharp contrast to the results of the aforementioned self-report-based studies.

Notwithstanding this clear contradiction, only a few studies (for example, Littel et al. [46], Azizi et al. [47]) have attempted to reconcile this conflict between SRA- and BE-based studies. Interestingly, these conflicting results from two methods seem to be cited rather selectively by follow-up or related studies in order to support their own argument, and therefore are likely to contribute to the serious babelization in this field. For this reason, we propose an integrated, multimodal approach combining assessments based on existing SRA and BE. First, we combine five representative questionnaires commonly used to assess or diagnose the psychological states of video game players (see Materials and Methods for details). The results of these assessments will be compared with the result of antisaccade experiment, a widely used behavioral measure of response inhibition [48], obtained from the same participants. The correlation between self-report scores and antisaccade performance measures will be analyzed using multimodal statistical analysis. Through this approach, our study aims to bridge the gap between the findings of two different research frameworks and to provide a unified view of the neurocognitive effect of video games and its implications.

## 2. Materials and Methods

### 2.1. Participants

Naive and young participants (*n* = 30, 10 females, age = 25.3 ± 2.6 years, range = 21–33) were recruited from the Korea Institute of Science and Technology (KIST) and surrounding university through KIST’s recruitment announcement. Participants were divided into two groups based on their video gaming frequencies [37]. Those who have been playing video games for more than one hour per week over a year were categorized as Video Game Player (VGP, *n* = 18, 1 female) group and the rest were categorized as Non-Video Game Players (NVGP, *n* = 12, 9 females). On average, video game play time of the VGP group was 6.78 ± 3.9 h per week, while that of the NVGP group was 0.04 ± 0.14 h per week. There was no significant age difference between the two groups (*p* = 0.798, Wilcoxon–Mann–Whitney test). The experiment was conducted in the Korea Institute of Science and Technology and the institutional ethics board has approved the study (IRB No. 2018-016).

### 2.2. Saccade Experiment

#### 2.2.1. Experimental Setup

Participants were seated comfortably on a chair and looked at a high-speed LED screen (120 Hz refresh rate) in front. The screen was 32 inches in size and placed 70 cm away from the participants, and the height of the chair was adjusted so that its center aligned to the participant’s eye level. To prevent head movement, the participant’s head was fixed using a custom-made chin rest. The experiment was conducted in a dark, soundproofed room, where no light source other than the screen was available.

#### 2.2.2. Experimental Design

**Figure 1:**
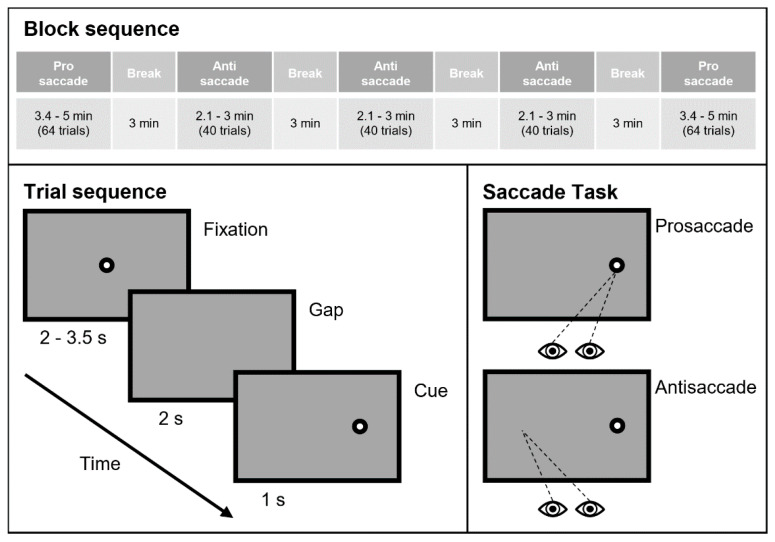
Saccade task protocol. The experiment consisted of five blocks of trials, in the order of pro, anti, anti, anti, pro. In each trial, the target appeared for 2 to 3.5 s in the center of the screen for fixation, then disappeared for 0.2 s (gap period). Then the circle randomly appeared again on the left or right side of the screen, and after 1 s, went back to the center and repeated the entire sequence. A detailed description of the Saccade task’s judgment and measurement is given in the next subsection.

Stimuli were presented using MATLAB Psychophysics Toolbox Version 3 [49]. Fixation target was displayed as a black circle (radius = 0.6°) with a grey bull’s eye (radius = 0.18°) on a light-grey (RGB value 127, 127, 127) background. When a trial started, a target appeared at the center of the screen, and participants were asked to fixate on the target. The duration of the fixation for each trial was randomly sampled from a non-ageing distribution [50] in order to discourage participants’ prediction of the remaining time, but the minimum and maximum waiting time were set to be 2.0 and 3.5 s respectively for practicality. After the fixation period ended, the target disappeared for 0.2 s as a gap period [51,52], and reappeared either on the left or on the right side of the screen for 1.0 s until the end of the trial. The distances from the center to the reappeared target were 6.5 cm (13.3°). Figure 1 (bottom left) summarizes the trial design.

The experiment consists of two types of trials: prosaccade and antisaccade. In prosaccade trials, when the target jumped to the left or right, participants were asked to make an eye movement toward the new target position as soon as possible. In the antisaccade trials, participants were asked to make the same saccadic movement, but toward the mirrored position of the target. Both prosaccade and antisaccade trials are sketched in Figure 1 (bottom right). The whole experiment consisted of five blocks of prosaccade and antisaccade trials in total. Participants first performed sixty-four prosaccade trials (block 1), followed by three consecutive blocks of forty antisaccade trials (blocks 2–4) and another sixty-four prosaccade trials (block 5). For each block, the same number of left and right targets appeared in a randomized order. Participants took three minutes break in-between the blocks [50]. Overall, each participant completed a total of 248 trials, consisting of 128 prosaccade and 120 antisaccade trials (Figure 1, top). 

Throughout the experiment, the participant’s horizontal eye movements were recorded using an electrooculogram (EOG). Two electrodes were placed at both sides of the eye around the temple, with the anode on the left and cathode on the right, and the ground electrode was placed in the glabella. A commercial amplifier (ECG100C, IPS100C-1, BIOPAC, Goleta, CA, USA) and a data acquisition board (USB-6001, National Instruments, Austin, TX, USA) were used to record EOG with a sampling rate of 1 kHz.

#### 2.2.3. Data Processing

**Figure 2:**
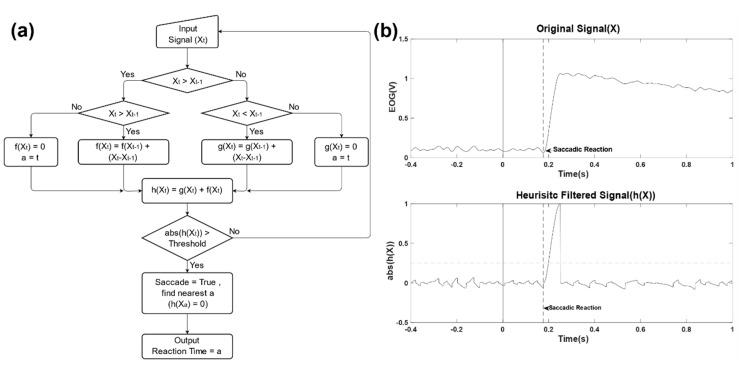
(**a**) Flow chart of the heuristic filter. Xt was noise removed signal from the original signal, h(Xt) was monotonic changed signal, and RT was Reaction Time when saccade occurred after target moved. (**b**) Example plots of original signal and heuristic filtered signal. The vertical solid line in original signal indicates when the target moved, and the vertical dotted line indicates RT. The horizontal dotted line in the heuristic filtered signal indicates a threshold for saccade detection, and the horizontal arrow indicates the point at which the saccadic reaction occurred.

Before analysis, EOG data were low pass filtered (Butterworth with a cutoff frequency at 30 Hz). Reaction time (RT) was determined by the time difference between the time when the target reappeared (time = 0) and the moment when a corresponding saccade was initiated. To avoid the effect of baseline drift commonly observed in EOG signals, a heuristic filter was used, similar to the ones used in previous studies [53,54]. In the heuristic filter, the signal is divided into a monotone increasing (f(X)) and monotone decreasing (g(X)), and then combined (h(X)). If heuristic filtered signal (h(X)) exceeds Threshold (±0.2, fixed value), it is determined that saccade occurred (see Figure 2a). Then, in order to find RT, we found the nearest point where monotone increasing and monotone decreasing intersect (h(X) = 0) from the point where saccade occurred (see Figure 2b). At this time, the left and right saccades are judged through a positive and negative value of EOG.

If a saccade was made toward the opposite direction of the target, it was classified as a direction error trial. The frequency of direction error trials over the experiment is quantified as an error rate [55]. Some trials were excluded from the statistics as outliers if saccade detected within 50 ms after the target moved, or if no saccade could be detected within 800 ms [51]. These excluded trials were 4.18 ± 4.19% over the total participants, and the difference between VGP (5.35 ± 4.48%) and NVGP (2.42 ± 3.11%) was not significant (*p* = 0.065, Wilcoxon–Mann–Whitney test). In the prosaccade task, the prosaccade error rate was too low to give meaningful statistics.

### 2.3. Self-Report Surveys

Five surveys have been measured for analysis based on the SRA framework: Brief Self-Control Scale (BSCS), Behavioral Activation System and Behavioral Inhibition System (BIS/BAS), Barratt Impulsiveness Scale (BIS-11), Beck Depression Inventory (BDI-II), Beck Anxiety Inventory (BAI). The BSCS is a measure of individual differences in self-control [56]. BSCS consists of 13 items on the 5-point Likert scale from 1 (strongly disagree) to 5 (strongly agree). The BIS/BAS measure two motivational systems that control behavior and contribute to determining human personality [57]. BIS/BAS consists of 20 items on the 4-point Likert scale from 1 (very true for me) to 4 (very false for me). In order to measure impulsivity, BIS-11-R [58], which is the revised version of BIS-11 [59], was used. BIS-11-R consists of 30 items on the 4-point Likert scale from 1 (very true for me) to 4 (very false for me). The scale is scored to a total score, three second-order factors, and six first-order factors. In this study, we use the total score. BDI-II is a revision of the BDI [60], and it has 21 items on the 4-point Likert scale from 0 (Not at all) to 3 (it bothered me a lot). If the total score is high, it can be diagnosed as having severe depressive symptoms. BAI [61] has 21 items on the 4-point Likert scale from 0 (Not at all) to 3 (it bothered me a lot). For all surveys, higher total scores indicate more severe symptoms.

## 3. Results

### 3.1. Behavioral Experiment Performance Measurements

#### 3.1.1. Reaction Time (RT)

**Figure 3:**
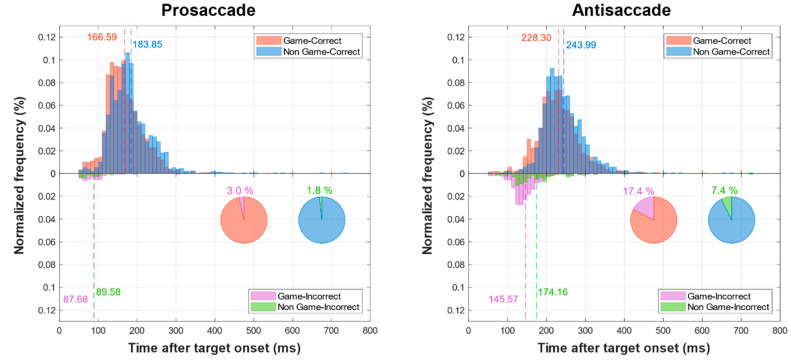
Normalized frequency histograms of the prosaccade (**left**) and the antisaccade (**right**) task. Histograms above the zero-line represent the RT distribution of correct trials, and those below represent incorrect trials. The distributions of RT of the VGP group are shown in red (correct) and purple (incorrect), and those of the NVGP group are shown in blue (correct) and green (incorrect). Dotted lines indicate mean RT values. Pie charts in the lower right corner summarize the error rates of VGP (**left**) and NVGP (**right**) group, respectively.

The difference in prosaccade RT between VGP and NVGP was statistically tested by student’s *t*-test. As shown in Figure 3 and Table 1, when comparing VGP (166.59 ± 28.48 ms) and NVGP (183.85 ± 29.90 ms), they did not show a significant difference (*p* = 0.122). The difference in antisaccade RT between VGP and NVGP was statistically tested by student’s *t*-test. When comparing VGP (228.30 ± 35.48 ms) and NVGP (243.99 ± 31.62 ms), they did not show a significant difference (*p* = 0.226). In addition, prosaccade RT was significantly faster than antisaccade RT for both VGP (*p* < 0.001) and NVGP (*p* < 0.001).

#### 3.1.2. Error Rate

The error rate of prosaccade task did not show normality, but the error rate of antisaccade task show normality. Therefore, the prosaccade error rate between VGP and NVGP was compared by Wilcoxon–Mann–Whitney test, and the antisaccade error rate was compared by student’s *t*-test. As shown in Figure 3 and Table 1, the result showed that the error rate of VGP (3.4 ± 2.42%) in the prosaccade test is not significantly different (*p* = 0.085) from that of NVGP (1.78 ± 1.76%), while the error rate of VGP (17.42 ± 12.33%) in the antisaccade test is significantly higher (*p* = 0.005) than that of NVGP (7.37 ± 4.69%).

### 3.2. Self-Report Surveys

#### 3.2.1. Brief Self-Control Scale (BSCS)

The self-report surveys’ score of BSCS in VGP, NVGP was statistically compared by student’s *t*-test. As shown in Table 1, when comparing VGP (37.66 ± 4.58) and NVGP (36.92 ± 5.42) in BSCS’s score, they did not show any significant difference (*p* = 0.686).

#### 3.2.2. Behavioral Activation System and Behavioral Inhibition System (BIS/BAS)

In BIS/BAS, self-report surveys’ total scores of BIS and BAS were statistically compared by student’s *t*-test. As shown in Table 1, when comparing VGP (15.5 ± 3.17/26.22 ± 5.09) and NVGP (18 ± 2.09/26.33 ± 5.19) in BIS/BAS’s total scores, BIS showed a significant difference (*p* = 0.023), however, BAS did not show a significant difference (*p* = 0.954).

#### 3.2.3. Barratt Impulsiveness Scale (BIS-11-R)

In BIS-11-R, self-report surveys’ score was statistically compared by student’s *t*-test. As shown in Table 1, when comparing VGP (61.33 ± 10.85) and NVGP (59.75 ± 11.09) in BIS-11-R’s score, they did not show a significant difference (*p* = 0.701).

#### 3.2.4. Beck Depression Inventory (BDI-II)

In BDI-II, self-report surveys’ score was statistically compared by student’s *t*-test. As shown in Table 1, when comparing VGP (10.39 ± 7.06) and NVGP (8.83 ± 5.89) in BDI-II’s score, they did not show a significant difference (*p* = 0.534). There was no participant over the cutoff score of severe depression [62]. 

#### 3.2.5. Beck Anxiety Inventory (BAI)

In BAI, self-report surveys’ score did not show normality. Therefore, the score was compared by Wilcoxon–Mann–Whitney test. As shown in Table 1, when comparing VGP (5.83 ± 6.11) and NVGP (5.42 ± 3.6) in BAI’s score, they did not show a significant difference (*p* = 0.671). There was no participant over the cutoff score of severe anxiety [63]. 

### 3.3. Correlation Analysis of the Behavioral Experiment Performance Measurements and the Self-Report Surveys

Pearson correlation analysis was conducted to investigate the correlation between the performance measures of the saccade tasks (RT and error rate) and the results of the self-report surveys. As shown in Table 2, no significant correlation was observed between the measurements of the saccade task and the score of the self-report surveys. 

In the correlation between variables in the saccade task, highly significant correlations were observed between the prosaccade RT and the antisaccade RT for all groups (all participants: *r* = 0.74, *p* < 0.001, VGP: *r* = 0.72, *p* < 0.001, NVGP: *r* = 0.73, *p* = 0.007). There was also a significant correlation between the prosaccade RT and antisaccade error rate for all participants and VGP groups (all participants: *r* = −0.5, *p* = 0.005, VGP: *r* = −0.55, *p* = 0.017), but not for NVGP group (NVGP: *r* = −0.15, *p* = 0.647). As shown in Table 2, the antisaccade RT and antisaccade error rate did not have a significant overall correlation.

In the correlation analysis between variables in the self-report, the significant correlation was observed for all participants group between BSCS and BIS-11-R (*r* = 0.49, *p* = 0.006), BSCS and BIS (r = −0.48, *p* = 0.008), BIS-11-R and BDI-II (*r* = 0.4, *p* = 0.029), BIS-11-R and BIS (*r* = −0.42, *p* = 0.02), BIS-11-R and BAS (*r* = −0.43, *p* < 0.018), BDI-II and BAI (*r* = 0.43, *p* = 0.019), BIS and BAS (*r* = 0.46, *p* = 0.01). Specifically, there was significant correlation BIS and BAS for VGP and NVGP groups (VGP: *r* = 0.48, *p* = 0.041, NVGP: *r* = 0.58, *p* = 0.049), while in contrast, there was no significant correlation between BIS-11-R and BDI-II (VGP: *r* = 0.4, *p* = 0.096, NVGP: *r* = 0.38, *p* = 0.226), BIS-11-R and BAS (VGP: *r* = −0.42, *p* = 0.083, NVGP: *r* = −0.45, *p* = 0.144), and BDI-II and BAI (VGP: *r* = 0.4, *p* = 0.104, NVGP: *r* = 0.53, *p* = 0.076) for VGP and NVGP groups. The correlation analysis between BSCS and BIS-11-R maintained a significant correlation in NVGP group but disappeared in VGP group (VGP: *r* = 0.34, *p* = 0.163, NVGP: *r* = 0.67, *p* = 0.018). In BSCS and BIS, significant correlation was maintained in VGP group but disappeared in NVGP group (VGP: *r* = −0.5, *p* = 0.037, NVGP: *r* = −0.54, *p* = 0.071). In BIS-11-R and BIS, significant correlation in VGP group was maintained but disappeared in NVGP group (VGP: *r* = -0.52, *p* = 0.029, NVGP: *r* = −0. 27, *p* = 0.395) (Table 2).

## 4. Discussion and Conclusions

To understand and reconcile the prominent discrepancies in evaluating the cognitive effect of video game playing, the present study directly compared the results of two representative frameworks (BE and SRA) that are widely used in the literature. As a typical test of the BE framework, we used the pro- and antisaccade task and statistically analyzed whether the video game playing has an effect of altering the RT and error rate of the saccadic tasks. For SRA, the same participants also completed five standardized self-report surveys that are normally used in the previous studies, with the same purpose of testing whether and how the video game playing affects the survey scores. As far as we are aware, this research is among few studies that directly compared, within and across participants, the effect of video game playing on the results of two different test frameworks and their correlations.

### 4.1. Behavioral Experiment Analysis on the Cognitive Effect of Video Game Playing

First, the VGP group showed significantly higher error rates during antisaccade trials than NVGP as shown in Figure 3 and Table 1. It is commonly believed that normal saccades are a mixture of two different types: (1) express saccades, fast reflexive saccades triggered by a direct stimulus–response mapping, and (2) regular saccades, slower saccades managed by a higher attentional control [48]. Since express saccades are less affected by higher-level task instructions and therefore are always triggered toward the stimulus, they should be effortfully inhibited during the antisaccade trials. For this reason, the higher number of erroneous antisaccades in the VGP group implies that VGPs are likely to generate more express saccades, or are likely to have greater difficulty inhibiting express saccades. In addition to the higher antisaccade error rates observed in VGPs, this interpretation is also supported by the following correlation analysis between saccade tasks, revealing a significant negative correlation between the prosaccade RT, where shorter RT implies a larger number of express saccades, and the antisaccade error rate in VGP, but not in NVGP. Therefore, the observed higher error rates of VGP groups can be interpreted as their weaker abilities in inhibitory control or, to some extent, an impulsivity problem [64].

Our result on the antisaccade error rate agrees well with the results of a group of studies on the impulse control characteristics of VGPs [46,65,66], which supported the idea that VGPs suffer an increased level of impulsivity [37,46,47]. This is also confirmed by neuroimaging studies demonstrating that video game playing can adversely affect regions in the frontal lobe that are involved in impulse control [6,7] and other mental functions related to IGD [67,68,69]. On the other hand, it is also important to point out that our result clearly dissents from another group of studies claiming that video game playing may improve perceptual–cognitive skills without any negative effect on the impulsiveness [34,37,39,70]. Therefore, our result of the saccadic experiment supports the idea that video game playing has a detrimental effect on cognition, and the effect is related to impulsivity.

While antisaccade error rates were different between VGPs and NGVPs, neither pro- nor antisaccade RTs showed any significant difference between the two groups, although there was a marginal trend toward faster RT for the VGP group. Therefore, our result of RT analysis does not agree with those from the literature that reported significantly faster RTs of VGPs [37,47]. It should be noted, however, that such discrepancy could have been simply caused by differences in task difficulty or data processing method; compared to our pro- and antisaccade tasks that followed rather standardized protocols in neuroscience studies [50], both pro- and antisaccade experiments used by Mack and Ilg [37] were substantially more difficult, including mixed pro- and antisaccade conditions within the same block with four targets (instead of two in our study) and additional double-step requirement. Indeed, the RTs detected by Mack and Ilg [37] tend to be substantially longer than the known RTs of human pro- and antisaccade [71]. Additionally, while the control group’s antisaccade error rate in our study was 7.4%, which is within the range of normally reported error rates [48], those reported by in Mack and Ilg [37] were noticeably higher, ranging from 30% to 40%. It is, therefore, possible that the greater task difficulty of their study might have an effect of amplifying the marginal trend observed in our study. Interestingly, the follow-up study by the same group [38] suggested that the observed effect of video game playing is likely to be caused by the change in higher cognitive processes, which supports the idea that the difference in RTs may become more prominent in cognitively more demanding tasks. In terms of the data processing method, it is important to point out that Mack and Ilg [37] included RTs of erroneous antisaccades when analyzing the prosaccade RT. Since erroneous antisaccades are likely to be express saccades and therefore are likely to have shorter RTs, this extra data processing may have an effect of altering the statistics of RT. The study by Azizi et al. [47] includes only a small number of antisaccade trials (60 trials for each participant), which makes it difficult to be compared with our result.

Apart from the video game playing studies, another point to notice is that our results fit very well with the result of studies on the Attention Deficit Hyperactivity Disorder (ADHD). A group of studies focused on how ADHD affects the saccadic performance showed that ADHD only affects the error rate but not RT [48,72,73], which is identical to our findings. This link between ADHD and video game playing can be easily speculated because ADHD is known to be primarily related to deficits in inhibitory control [74], and also it was confirmed by neuroimaging studies that ADHD impedes the activation of inhibitory brain areas such as the dorsolateral prefrontal cortex or superior parietal lobe [75], which play central roles in saccadic control. This raises a new research question of investigating the potential of using BEs to understand the relationship between video game playing and ADHD.

### 4.2. Self-Report Survey Analysis on the Cognitive Effect of Video Game Playing

The result of SRA on the same set of participants showed that VGP has significantly lower BIS scores than NVGP, while no difference between VPG and NVGP was observed in the other four surveys (see Table 1). Regarding the BIS/BAS scores, Quay [76,77] argued that high BAS scores would trigger extreme responses to reward signals, contributing to the development of aggressive behaviors such as behavioral disorders, and low BIS scores will contribute to the development of ADHD due to their low sensitivities of punishment clue associated with anxiety. In addition, Wang et al.’s study [78] interpreted that the higher the BIS score indicates the higher behavioral inhibition and predisposing characteristic of social anxiety [79,80,81]. Based on these interpretations, the low BIS scores in our SRA results correlated with low behavioral inhibition and ADHD, which may be interpreted as correlated with the above BE results. 

However, the lower BIS score in our study is different from other studies that show a higher BIS score [78] and a similar BIS score [21,82,83] compared to the standard BIS score. Further investigation would be needed in order to understand this conflict, but it is likely that such discrepancies were caused by the difference between selection criteria; while VGPs in BE-based studies [34,37,47], including ours, were classified based on video game usage time, VGPs in the SRA-based studies [21,78,82,83] were classified through questionnaires used to identify gaming-related mental disorders, indicating that more severe criteria were applied for SRA-based studies. 

Indeed, in the study of Wang et al. [78], where the BIS score of the IGD group was significantly higher than the control group, the average weekly game play time significantly exceeded the other studies [21,82,83] that did not show a difference [78]: average 40 h, Park et al. [82]: 83.2% of participants do not exceed 8 h, Zvyagintsev et al. [83]: average 5 h, Na et al. [21]: average time does not exceed 10 h, and in our study: average 6.78 h). This can be seen in the results of other survey scales; in a study [23] using the BSCS scale, Chen and Leung [23] used BSCS to assess self-control, which indicated that self-control was associated with mobile game addiction but not mobile game use. Studies [20,84] using other surveys, including BIS-11, have also focused on identifying addicts by IGD.

In addition, only 22% of the VGP (4 out of 18) group in our study corresponded to the IGD of the DSM-5 and, based on our case, it is reasonable to assume that a large portion of VGPs recruited in the previous BE-based studies could be categorized as NVGP in the SRA-based studies. And considering the study [85,86,87] that only a small group of general gamers are IGDs, this implies that the main controversy between BE- and SRA-based studies on the cognitive effect of video game playing can potentially originate from the nonlinearity of participant, which requires further investigation. 

### 4.3. Correlation Analysis between Behavioral-Experiment-Based and Self-Report-Based Analysis

Although both our BE and SRA consistently showed the cognitive effect of video game playing, the following correlation analyses in Table 2 have revealed that there was no significant correlation between the antisaccade task and the BIS score. In fact, such lack of correlation has also been participants of controversy in the previous studies [88,89,90,91]; while there are studies that found correlation [88] in the antisaccade error rate and the overall score of BIS-11, or only in the subscale of BIS-11 [89,91], other studies did not find any correlation including additional subscale of BIS-11 [90]. Similar to our argument above, it was pointed out that such discrepancy can possibly be due to a complex interaction between the participant recruitment criteria and the survey score [90]. Based on this interpretation, our result implies that no elements of BIS-11 could affect the antisaccade error rate in the VGP group. 

Regarding the correlation analyses to the survey scores other than BIS, the results of the correlation analysis of BSCS and the saccadic parameters, which did not have a significant result, are consistent with the literature, suggesting that there was no negative transfer of executive control to the performance of antisaccade [92]. In addition, whereas previous studies have shown that depression or anxiety disorders affect the saccade task [93], the lack of correlations between saccadic parameters and the survey scores related to depression or anxiety disorders, BDI-II and BAI in our case, is understandable since no participant either in the VGP or in the NVGP group has exceeded the cutoff scores for those disorder criteria. 

**Figure 4:**
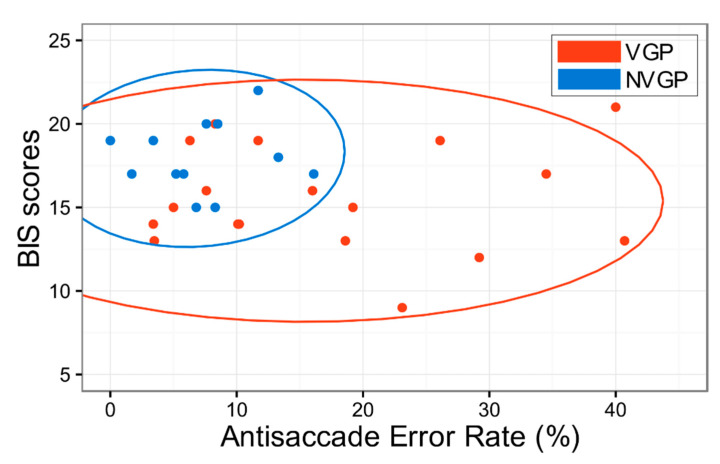
The scatter plot with 95% confidence ellipse for antisaccade error rate and BIS scores. VGP and NVGP were represented in red and blue. BIS: Behavioral Inhibition System.

Notwithstanding the conflict in literature, it is still worthy to speculate on possible reasons for such decorrelations. First, it is noticeable that the variances of the antisaccade error rate in VGP and NVGP are significantly different (*p* = 0.002, Levene’s Test) as shown in Figure 4. The higher variance of VGP’s antisaccade error rate suggests that the effect of video game playing on the saccadic task could be highly heterogeneous, affecting only a portion of participants in the VGP group. This implies that the criteria used by existing studies, including ours, to classify VGPs based on the frequency of video game playing could be highly oversimplifying the possibly multi-dimensional nature of the video game’s cognitive effects, ignoring potential effects of the other parameters, such as the genre [21] or the contents [83] of the game. 

There was some limitation of our study is the difference in gender ratio between groups. In fact, the lack of gender balance, i.e., a male dominancy, is one of the typical aspects characterizing the video game population [94]. This does not only make it practically difficult to recruit video game players while maintaining the gender balance, but also indicate that enforcing the gender balance of the participants could potentially distort the true characteristics of the video game player population. For these reasons, we did not consider gender balance like previous studies [37,47] and relied on a random selection process to eliminate methodological flaws [95]. This resulted in a pronounced unbalance in gender between our participant groups: the percentage of females in the NVGP group (75%) was substantially higher than that in the VGP group (6%). Although previous studies [58,96,97,98] consistently showed no gender differences, there are also studies suggesting the gender difference in the survey score [99,100] and the effect of video game playing [101,102,103], which remain future consideration. As with gender bias, some studies [104,105,106] have shown that caffeine has little effect on oculomotor testing, but the lack of screening for caffeine ingest by participants prior to the study should be supplemented in future studies.

In summary, our results of BE and SRA support the connection between video game and impulsivity. However, VGP’s distribution of antisaccade error rate is higher, and there is no correlation between the results of BE and SRA, suggesting that the cognitive effects of video games depend on a complex interplay between psychological and neurocognitive processes, and that the effects and domain vary widely from person to person. With these findings, the result of our study support the necessity of incorporating multimodal approaches to assess the effect of video game play.

## Figures and Tables

**Table 1:** Verification of statistical differences between VGP and NVGP.

Scale	VGP (*n* = 18)	NVGP (*n* = 12)	*p*-Value	Effect Size (Cohen’s d)
Prosaccade RT (ms)	166.6 ± 28.5	183.9 ± 29.9	0.122 (t)	0.5943
Antisaccade RT (ms)	228.3 ± 35.5	244 ± 31.6	0.226 (t)	0.4614
Prosaccade Error Rate (%)	3.4 ± 2.42	1.78 ± 1.76	0.085 (W)	0.7440
Antisaccade Error Rate (%)	17.4 ± 12.3	7.4 ± 4.7	0.005 ** (t)	1
BSCS	37.7 ± 4.6	36.9 ± 5.4	0.686 (t)	0.1523
BIS/BAS	15.5 ± 3.2/26.2 ± 5.1	18 ± 2.1/26.3 ± 5.2	0.023 */0.954 (t)	0.8949/0.0216
BIS-11-R	61.3 ± 10.8	59.7 ± 11.1	0.701 (t)	0.1447
BDI-II	10.4 ± 7.1	8.8 ± 5.9	0.534 (t)	0.2349
BAI	5.8 ± 6.1	5.4 ± 3.6	0.671 (W)	0.0791

RT: Reaction Time, BSCS: Brief Self-Control Scale, BIS: Behavioral Inhibition System, BAS: Behavioral Activation System, BIS-11-R: Barratt Impulsiveness Scale, BDI-II: Beck Depression Inventory, BAI: Beck Anxiety Inventory. * *p* < 0.05, ** *p* < 0.01, t: Student’s *t*-test, W: Wilcoxon–Mann–Whitney test.

**Table 2:** Correlation Matrices of all participants, VGP, and NVGP.

Group All Participant									
	**Pro RT**	**Anti RT**	**Anti Error**	**BSCS**	**BIS-11-R**	**BDI-II**	**BAI**	**BIS**	**BAS**
Pro RT	-	0.74 ***	−0.50 **	0.11	0.22	0.06	−0.20	0.03	−0.03
Anti RT	-	-	−0.34	0.07	0.29	0.07	−0.09	−0.05	−0.18
Anti Error	-	-	-	0.24	0.16	0.26	0.19	−0.16	0.03
BSCS	-	-	-	-	0.49 **	0.22	0.06	−0.48 **	−0.12
BIS-11-R	-	-	-	-	-	0.40 *	0.23	−0.42 *	−0.43 *
BDI-II	-	-	-	-	-	-	0.43 *	−0.26	−0.24
BAI	-	-	-	-	-	-	-	−0.10	−0.20
BIS	-	-	-	-	-	-	-	-	0.46 *
BAS	-	-	-	-	-	-	-	-	-
**Group VGP. Above Main Diagonal**									
**Group NVGP. Below Main Diagonal**									
	**Pro RT**	**Anti RT**	**Anti Error**	**BSCS**	**BIS-11-R**	**BDI-II**	**BAI**	**BIS**	**BAS**
Pro RT	-	0.72 ***	−0.55 *	0.05	0.04	0.04	−0.29	−0.07	−0.05
Anti RT	0.73 **	-	−0.40	−0.01	0.33	0.10	−0.10	−0.12	−0.29
Anti Error	−0.15	0.21	-	0.18	0.12	0.22	0.19	0.03	0.10
BSCS	0.25	0.25	0.53	-	0.34	0.02	−0.03	−0.50 *	0.15
BIS−11-R	0.57	0.28	0.30	0.67 *	-	0.40	0.18	−0.52 *	−0.42
BDI-II	0.23	0.11	0.31	0.52	0.38	-	0.40	−0.28	−0.37
BAI	0.04	−0.02	0.25	0.27	0.36	0.53	-	−0.11	−0.41
BIS	−0.19	−0.29	0.08	−0.54	−0.27	−0.12	−0.02	-	0.48 *
BAS	−0.01	−0.01	−0.19	−0.47	−0.45	0.00	0.34	0.58 *	-

Pro RT: Prosaccade Reaction Time, Anti RT: Antisaccade Reaction Time, Anti Error: Antisaccade Error Rate, BSCS: Brief Self-Control Scale, BIS-11-R: Barratt Impulsiveness Scale, BDI-II: Beck Depression Inventory, BAI: Beck Anxiety Inventory, BIS: Behavioral Inhibition System, BAS: Behavioral Activation System. * *p* < 0.05, ** *p* < 0.01, *** *p* < 0.001.
